# Methotrexate, Hydrocortisone, Vincristine, Sobuzoxane, and Etoposide Is an Effective Option for Relapsed T-cell Prolymphocytic Leukemia with Loss of CD52 Expression after Retreatment with Alemtuzumab

**DOI:** 10.31662/jmaj.2024-0145

**Published:** 2024-09-27

**Authors:** Shohei Ikeda, Manabu Suzuki, Masumi Sukegawa, Saburo Tsunoda, Masatsugu Ohta

**Affiliations:** 1 Department of Hematology, Fukushima Medical University Aizu Medical Center, Aizuwakamatsu, Japan

**Keywords:** T-cell prolymphocytic leukemia (T-PLL), MTX-HOPE, Alemtuzumab

## Abstract

T-cell prolymphocytic leukemia (T-PLL) is a rare and highly aggressive mature T-cell neoplasm. Although the response rate to alemtuzumab, an anti-CD52 antibody, is high, it is difficult to cure the disease with this agent alone. Therefore, hematopoietic stem cell transplantation is recommended for eligible patients. However, there are few effective salvage therapy options for patients ineligible for hematopoietic stem cell transplantation. In this study, we report the case of an elderly patient with relapsed or refractory T-PLL who underwent salvage therapy with methotrexate, hydrocortisone, vincristine, sobuzoxane, and etoposide (MTX-HOPE).

The patient was an 85-year-old man. He was administered alemtuzumab twice (at the time of initial treatment and relapse) and cyclophosphamide, vincristine, and hydrocortisone chemotherapy. Furthermore, novel therapeutic drugs (venetoclax and tofacitinib) were administered based on previous case reports. However, the patient was resistant to all treatments, and the tumor cells lost CD52 expression. We administered MTX-HOPE, and the patient survived for approximately 8 months. Although red blood transfusions were necessary because of disease progression, no adverse events were observed because of treatment, and the patient was able to maintain activities of daily living until immediately before death.

MTX-HOPE is a combination of classical chemotherapy agents originally developed for palliative chemotherapy in frail patients with refractory lymphoma. MTX-HOPE has been reported to be effective against T-cell tumors. Severe nonhematologic adverse events are rarely reported; however, bone marrow suppression is commonly observed. Grade 3-4 neutropenia has been documented in approximately half of the patients. Therefore, patients should be closely monitored, particularly at the onset of therapy. Consideration should be given to suspending treatment, adjusting the administration interval, or administering G-CSF if necessary. The treatment interval can be appropriately adjusted, making it a valuable treatment option for refractory T-PLL.

## Introduction

T-cell prolymphocytic leukemia (T-PLL) is a rare and highly aggressive T-cell neoplasm ^(1)^. Alemtuzumab, a humanized anti-CD52 monoclonal antibody, is effective against T-PLL, with an overall response rate of approximately 80%. Given the high relapse rate within 12 months, stem cell transplantation remains the only curative option for this disease ^(2)^. The prognosis is poor, with a median overall survival (OS) and a 1-year OS of 1.2 years and 56%, respectively ^(1)^. Salvage therapies for patients with relapsed or refractory T-PLL ineligible for transplantation are severely limited, and outcomes have not significantly improved over the past two decades. In this study, we report a patient who exhibited a favorable response to methotrexate, hydrocortisone, vincristine, sobuzoxane, and etoposide (MTX-HOPE) after retreatment with alemtuzumab ^(3)^.

## Case Report

**Figure 1.**
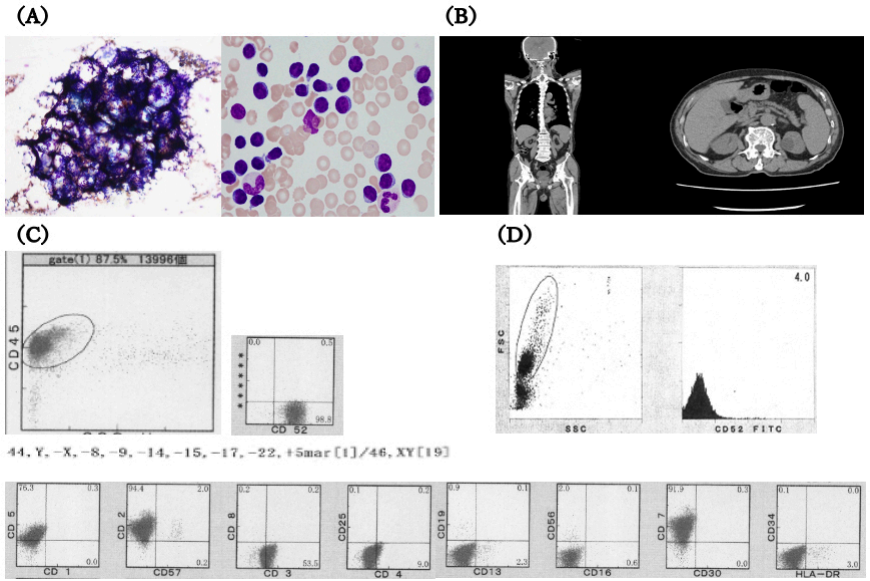
Results of laboratory testing at the first visit were as follows: (A) bone marrow smear showing cellular proliferation; (B) coronal and sagittal CT revealing splenomegaly, but no lymphadenopathy is observed; (C) flow cytometry showing tumor cells positive for CD2, CD3, CD5, CD7, and CD52 but negative for CD1, CD4, CD8, CD13, CD16, CD19, CD25, CD30, CD34, CD56, CD57, and HLA-DR. G-banding revealed complex chromosomal rearrangements. (D) CD52 in lymphocytes decreased to an extremely low level (4.0%) during relapse.

An 85-year-old man was referred to our hospital for further hyperleukocytosis examination. Laboratory tests revealed significant leukocytosis, anemia, and thrombocytopenia (white blood cell [WBC] count 34.2 × 10^4^/μL, Hb 10.2 g/dL, platelet 6.2 × 10^4^/μL) with 94% prolymphocytes. Bone marrow examination showed hypercellularity. Flow cytometry and immunohistochemistry of abnormal lymphocytes showed positivity for CD2, CD3, CD5, CD7, and CD52 and negativity for CD1, CD4, CD8, CD13, CD16, CD19, CD25, CD30, CD34, CD56, CD57, and HLA-DR. HTLV-1 antibodies were negative. The aforementioned findings led to the final diagnosis of T-PLL. CT revealed splenomegaly (Figure 1A, 1B, and 1C). Within 1 month, the disease rapidly worsened, and the patient’s performance status declined from 1 to 4 because of general fatigue and loss of appetite.

We administered a combination of low-dose conventional chemotherapy in addition to alemtuzumab. The WBC count began to decrease 15 days after administration, and the patient completed 12 weeks of therapy. Subsequent bone marrow examination confirmed complete remission.

T-PLL relapsed approximately 14 months after the initiation of alemtuzumab therapy. The patient was treated with venetoclax and tofacitinib based on previous reports ^(4), (5)^. However, these treatments were ineffective.

Because 99% of the abnormal lymphocytes were positive for CD52 expression, the patient underwent alemtuzumab retreatment. Post-treatment bone marrow analysis revealed residual tumor, and 1 month later, relapse occurred. Notably, the tumor cells had lost CD52 expression (Figure 1D).

**Figure 2.**
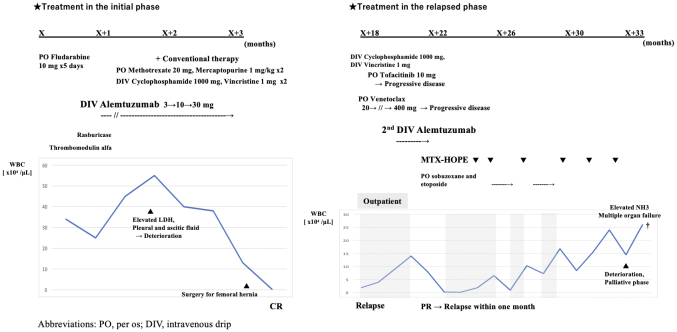
Clinical course of the initial and relapse phases.

The tumor cells were resistant to multiple drugs, and the patient’s prognosis was extremely poor. We introduced MTX-HOPE in an outpatient setting. Dosing intervals were adjusted at 2-4 weeks, and vincristine was temporarily omitted to reduce the risk of peripheral neuropathy and the burden of hospital visits. Social factors forced the patient to be hospitalized. MTX-HOPE was continued for approximately 8 months after its introduction; however, the patient died approximately 33 months after diagnosis. He could control his tumor burden without any significant decline in ADL until immediately before his death. Although the patient required red blood transfusion therapy because of disease progression, treatment-related hematologic adverse events, bacterial and viral infections, and other nonhematologic adverse events were minimally managed (Figure 2).

## Discussion

MTX-HOPE is a treatment regimen developed by Tsunoda et al. ^(6)^. The treatment consists of two topoisomerase inhibitors with different mechanisms of action (sobuzoxane and etoposide), oral methotrexate, and low-dose hydrocortisone and vincristine (Table 1). Vincristine administration 8-24 h after methotrexate produces strong antitumor effects. Simultaneous administration of sobuzoxane and etoposide also has potent antitumor effects ^(7)^. Although each of these two drugs is administered at a low dose, their synergistic effects have been reported to reduce toxicity and ensure efficacy.

**Table 1.** MTX-HOPE Protocol.

Agent	Dose/day	Route	Days of administration
Methotrexate (MTX)	20 mg	po	1
Hydrocortisone (HC)	100 mg	div (15min)	2
Vincristine (VCR)	1 mg	div (15min)	2
Sobuzoxane (MST-16)	400 mg	po	3, 4
Etoposide (ETP)	25 mg	po	3, 4

Abbreviations: MTX-HOPE, methotrexate, hydrocortisone, vincristine, sobuzoxane and etoposide; po, per os; div, intravenous drip

MTX-HOPE was originally designed for older or frail patients with lymphoma who are ineligible for standard therapy. Fukunaga et al. and Suzuki et al. reported its usefulness in clinical practice ^(8), (9)^. In particular, in the study of Suzuki et al. of 42 patients with relapsed or refractory lymphoma, which included 11 patients with T-cell lymphoma, 60% of the patients had a performance status of ≥2, indicating frailty. Although the median OS of the 42 patients was 7 months, the 1- and 2-year OS rates were 43.7% and 40.8%, respectively, indicating the feasibility of long-term treatment ^(9)^.

Sobuzoxane and etoposide, which are crucial components of the MTX-HOPE regimen, and their combination therapy have been widely used for the treatment of T-cell lymphoma, particularly adult T-cell leukemia, and have demonstrated a certain level of therapeutic efficacy ^(10)^.

In conclusion, although MTX-HOPE is a classical chemotherapy, it may be an effective and safe treatment option for relapsed or refractory T-PLL.

## Article Information

### Conflicts of Interest

None

### Author Contributions

S.I., M.S., M.S., S.T., and M.O. were involved in data acquisition, analysis, and interpretation. S.I. drafted the manuscript. All authors have contributed to the revision of the manuscript and approved the final version.

### Approval by Institutional Review Board (IRB)

The patient’s son signed informed consent (generic consent forms) for the academic use of the data.

IRB Approval Code and Institution Name: Not applicable.
